# Physician Review Websites: Understanding Patient Satisfaction with Ophthalmologists Using Natural Language Processing

**DOI:** 10.1155/2023/4762460

**Published:** 2023-03-08

**Authors:** Jason J. Jo, Christopher P. Cheng, Stephanie Ying, James G. Chelnis

**Affiliations:** ^1^Department of Medical Education, Icahn School of Medicine at Mount Sinai, New York, NY 10029, USA; ^2^Manhattan Face and Eye, New York, NY 10019, USA

## Abstract

**Introduction:**

The presence and influence of physician review websites (PRW) have increased significantly in the field of medicine. This study aims to better understand determinants of patient satisfaction and the sentiment of ophthalmologists using natural language processing of Healthgrades reviews.

**Methods:**

Healthgrades is a PRW where patients submit verified reviews, containing a star rating and a narrative review, of US-based ophthalmologists. This was a quantitative observational study conducted on May 23, 2022. We identified associations between physician demographics and both the sentiment analysis scores of narrative reviews and star ratings using the Student's *t*-tests and one-way ANOVA tests. After natural language processing the reviews, a logistic regression explored the impacts of the most frequent words on the positivity of a given review.

**Results:**

This study examined a total of 16700 reviews of 1125 ophthalmologists. Ophthalmologists of younger age and male gender received statistically significantly higher star ratings and sentiment analysis scores; analysis of location of practice did not affect scores. Textual analysis revealed that words describing the physician's personality, such as “friendly” and “caring,” increased the likelihood of reviews being positive more than descriptors of the visit's effectiveness, such as “results” and “efficient.”

**Conclusion:**

Younger and male ophthalmologists received higher star ratings and sentiment analysis scores. Additionally, results indicated that words describing the ophthalmologist's pleasant personality and the visit's effectiveness most positively impacted a review, whereas descriptors of a wait or an unpleasant personality most negatively impacted a review.

## 1. Introduction

Over the last few decades, there has been a widespread increase in the utilization and reliance on the Internet and other digital technologies, a trend that has inevitably made its way into the healthcare landscape [1–3]. More recently, the surge in digital activity following the COVID-19 pandemic has pulled the relationship between healthcare and the Internet closer together [4]. Physician-rating websites (PRWs), which utilize patient reviews to rate, evaluate, and rank physicians, have grown in popularity as a way to gain valuable information about a physician before making a decision [5, 6]. It is estimated that at least one in six practicing physicians in the United States has been reviewed online, and prior survey studies suggest that these websites can have a significant impact on patient decision-making [7, 8]. For instance, Hanauer et al. [7] found that 35% of surveyed individuals chose a physician based on their positive ratings, and 37% chose against a physician based on their negative ratings. A deeper understanding of PRWs, their trends, and their true impact on patient decision-making is critical to ensuring that physicians understand both their public perception and their patients' sentiments.

Within the field of ophthalmology, there is a need for deeper exploration of the physician review and rating landscape. Some smaller scale studies have been performed. For instance, Skrzypecki and Przybek in 2018 analyzed the online ratings of 105 ophthalmologists who exemplified “outstanding scientific performance,” based on their overall number of citations or Hirsch index, and found that there was no correlation between these academic and scientific achievement metrics and patient ratings on Healthgrades (https://healthgrades.com) [9]. Within the subspecialty of Ophthalmic Plastic and Reconstructive Surgery, Vu et al. [10] looked at the Healthgrades evaluations of 612 US-based members of the American Society of Ophthalmic Plastic and Reconstructive Surgery (ASOPRS) and found that most ASOPRS surgeons had at least one rating on the website. While ratings were generally high, long wait times were correlated strongly with lower recommendation scores. Smith et al. performed a larger-scale study [11], in which over 80,000 online reviews were analyzed. They found that higher scores were seen in ophthalmologists, who added photographs or a short biography to their page, as well as those who had shorter wait times, younger physicians, and those without a history of malpractice claims. There remains the opportunity to further understand the PRW landscape within ophthalmology through more large-scale studies and also through investigating the nuances of patient reviews, such as with more advanced technologies like machine learning and natural language processing.

Using natural language processing (NLP), this study explores PRWs through the analysis of star ratings and sentiment analysis scores in association with demographics and patient-written review content. Its aim is to use computational analysis to learn what specific factors contribute to overall sentiment and ratings of ophthalmologists on PRWs. By gaining a better understanding of the most common reasons and sentiments behind a positive or negative PRW review, ophthalmologists may be better equipped to optimize their public-facing profile as well as provide more patient-centric care by adjusting to patient sentiment. Furthermore, the identification of certain qualities or demographics that lead to more positive sentiment may also elucidate bias within the platform and subsequently generate a deeper knowledge base for appropriate initiatives aimed at increased transparency.

## 2. Materials and Methods

### 2.1. Data Collection

Patient-written reviews of US-based ophthalmologists from the online PRW “https://healthgrades.com” were collected using web scraping [12] on May 23, 2022, which automates manual extraction of patient review data via a web browser. We selected Healthgrades for analysis in this quantitative observational study due to its popularity amongst physicians and patients as well as the fact that it was more permissive of web scraping compared to other physician review sites. In addition, Healthgrades includes all physicians who have active profiles on the National Provider Identifier Registry. The data collected consisted of physicians' demographic information (gender, age, and location of practice), narrative reviews (words/phrases used and average rating), and a star rating, which is scored out of five and is assigned to each physician based on the average scores of their patient-written reviews. Only patients with access to the Internet and an Internet-accessing electronic device were able to submit reviews to Healthgrades. We defined inclusion criteria to be as many physicians classified as an “Ophthalmologist” that Healthgrades allowed to be extracted with search location parameters set to “National.” The exclusion criteria omitted any reviews given to physicians with fewer than a total of six reviews.

### 2.2. Natural Language Processing (VADER Sentiment Analysis)

In this study, sentiment analysis is used to study the opinions, feelings, and views of patients about their physicians through computational analysis of Healthgrades written reviews. The Valence Aware Dictionary and sEntiment Reasoner (VADER) is a commonly used Python package in the field of NLP to gather sentiment analysis from social platforms [13]. The VADER model translates written phrases into normalized scores that represent the positivity or negativity of the sentiment, taking into account writing features such as punctuation, capitalization, and degree modifiers. It accomplishes this by first, converting each word to a sentiment “valence” score from negative four to positive four that accounts for polarity and intensity. Subsequently, these scores are summed and adjusted based on the general, grammatical, and syntactical rules as set by VADER. Finally, the scores are normalized to a compound score that ranges from a negative one to a positive one. We selected VADER given its past uses in online patient review analysis as well as its sensitivity to the language and format of social media platforms [14–16].

### 2.3. Data Analysis

We trained a linear regression model to describe the relationship between the average VADER score and the Healthgrades star rating of each sampled ophthalmologist. If the VADER sentiment analysis were valid according to the Healthgrades star rating, the graph would display a linear relationship between the two variables. The *R*-squared value of this model was also used to optimize the minimum number of reviews required per physician to be included in this study. This method in determining the exclusion criteria was also used in the studies by Tang et al. [14–16]. The Student's *t*-tests were used to evaluate relationships between gender and both sentiment analysis scores and star ratings. One-way ANOVA tests were used to compare age and location of practice with both sentiment analysis scores and star ratings; the directionality of significant results was validated with a Tukey test. Geographic subgroups were defined as Northeast, Southeast, Southwest, Midwest, and West [17]. Age subgroups were defined in increments of ten to optimize ease of analysis and evenness of distribution. Prior to any text analysis, we completed natural language preprocessing, which tokenized and removed stop words from the text. The word frequency analysis identified every unique word in the patient-written reviews and documented their frequencies. We repeated the same procedure for bigrams, or two-word pairs. Further, a multivariate logistic regression model quantified the most impactful words or bigrams in determining whether or not a review was positive, the cutoff for which was a VADER sentiment score greater than 0.5.

## 3. Results

Out of 23815 reviews web scraped from Healthgrades on May 23, 2022, inclusion and exclusion criteria produced a total of 16700 reviews of 1125 ophthalmologists in the United States for analysis. The gender identity, age, and geographic location of the physician were identified as reported on the website (Table 1).

### 3.1. Model Validation

**Figure 1:**
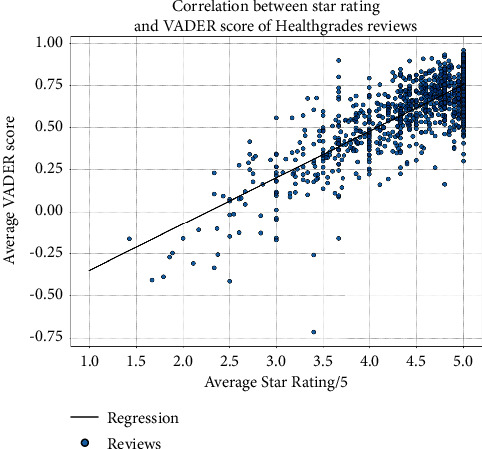
Average rating in comparison with average VADER sentiment analysis score in ophthalmologists included in analysis.

We tested the validity of VADER sentiment analysis scores using a linear regression between the average VADER score and the Healthgrades star rating. We found a positive correlation (*R*-squared = 0.647; *p* < 0.001), showing VADER scores to be largely in agreement with star ratings (Figure 1).

### 3.2. Gender, Age, and Location of Practice Analysis

We completed gender analysis of patient reviews the using Student's *t*-tests. Results indicated that male ophthalmologists received higher star ratings (4.61 v. 4.55; *p* < 0.001) than female ophthalmologists. The differences in sentiment analysis scores (0.645 v. 0.624; *p* < 0.002) between male and female ophthalmologists were also shown to be statistically significant (Table 2).

We completed age analysis by identifying average star ratings and sentiment analysis scores in four subgroups: <40, 40–49, 50–59, and >60. One-way ANOVA tests were run to study statistical differences between the two aforementioned markers. The differences in both star ratings (4.72 v. 4.51 v. 4.38 v. 4.40; *p* < 0.001) and sentiment analysis scores (0.691 v. 0.616 v. 0.586 v. 0.590; *p* < 0.001) between age subgroups were shown to be statistically significant (Tables 3–5).

We completed location analysis by separating ophthalmologists into five geographical subgroups: West, Midwest, Southwest, Southeast, and Northeast. We studied the relationship between average star ratings and sentiment analysis scores with location of practice using one-way ANOVA tests. Unlike gender and age, differences in star ratings (4.48 v. 4.46 v. 4.51 v. 4.50 v. 4.38; *p*=0.21), and sentiment analysis scores (0.623 v. 0.588 v. 0.634 v. 0.614 v. 0.582; *p*=0.12) between locations of practice were not shown to be statistically significant (Table 6).

### 3.3. Single-word and Bigram Frequency Analysis

Single-words and bigrams most frequently used in Healthgrades best and worst patient-written reviews were identified (Tables 7 and 8). Clinically irrelevant words and bigrams were omitted from this analysis. For instance, single-words such as “great” and “said” or bigrams such as “best,” “ophthalmologist,” “worst,” and “enemy” were frequently used, but proved irrelevant to understanding the reasons behind patient sentiment through linguistic analysis.

Of the included single-words, both the best and worst reviews had high frequencies of words describing the ophthalmologist's approach and atmosphere as well as the visit's effectiveness and efficiency. The best reviews included words such as “friendly,” “caring,” “kind,” and “comfortable” as well as “results,” “helpful,” and “efficient.” The worst reviews included words such as “rude,” “unprofessional,” “arrogant,” and “condescending” as well as “waiting,” “waited,” and “rushed” (Table 7).

Bigram analysis showed similar trends and similar words. Best reviews included high frequencies of the following: “friendly,” “helpful”; “kind,” “caring”; “friendly,” “efficient”; “truly,” “cares”; “great,” “results”; “staff,” “best”; “everyone,” and “friendly.” The worst reviews included high frequencies of the following bigrams: “never,” “return”; “refused,” “see;” “staff,” “unprofessional”; “waiting,” “hours”; “billed,” “insurance”; “condescending,” “rude”; “horrible,” and “bedside” (Table 8).

### 3.4. Multivariate Logistic Regression

A multivariate logistic regression was performed using clinically relevant words to determine the likelihood that a specific single-word or bigram would be included in a positive patient-written review (Table 9).

### 3.5. Spread of Star Scores

There was a total of 14620 five-star and 1458 one-star reviews, in contrast with 196 two-star, 140 three-star, and 286 four-star reviews (Table 10). One- and five-star reviews made up around 96% of total reviews, while two-, three-, and four-star reviews made up around 4%.

## 4. Discussion

Given the recent increase in popularity of PRWs in the past decade, it is important for ophthalmologists to better understand these platforms, which often serve as filter mechanisms for patients who are searching for a new ophthalmologist [5, 6, 18]. The present study investigates patient-written reviews of US ophthalmologists on the popular physician review website, Healthgrades. The statistical relationships between certain physician demographics and both star rating and average sentiment score were studied. Also, single-word and bigram frequencies were noted and analyzed via a multivariate logistic regression for their impact on determining whether or not a review was positive, i.e. having a VADER score > 5.

To the knowledge of the authors, only three studies in the field of ophthalmology have explored PRWs [9–11]. This study represents the second largest analysis of ophthalmology-specific PRWs, with 16700 reviews of 1125 ophthalmologists. It also represents the first study to use natural language processing to gauge patient sentiment from patient-written reviews, offering unprecedented granular insight into the impact of word choice on sentiment analysis outcomes, and thereby star rating outcomes. This study's results suggest that younger and male ophthalmologists tend to receive higher star ratings and reviews with higher sentiment analysis scores on Healthgrades. Moreover, the multivariate logistic regression indicated that being “confident,” “friendly,” and “caring” held a greater odds ratio in determining the positivity of a sentiment analysis score than outcome-pertinent diction such as “results” and “efficient.”

In contrast to the results reported by Vu et al. on ASOPRS surgeons visible on Healthgrades, this study found that male ophthalmologists received higher star ratings than female ophthalmologists on Healthgrades [10, 11]. Conversely, Smith et al. analyzed two PRWs, Healthgrades and Vitals, and determined no statistically significant difference between male and female ophthalmologists in star rating. Given the greater number and broader source of patient-written reviews that were analyzed by Smith et al., it is likely that PRWs in general do not indicate male ophthalmologists are more favorably received by patients compared with female ophthalmologists. However, it is of note that when we analyzed Healthgrades in isolation, we found the aforementioned trend to be statistically significant. Furthermore, sentiment analysis scores, which were not analyzed by previous studies, were also shown to favor male ophthalmologists on Healthgrades. As such, additional research should explore sentiment analysis score differences in male and female ophthalmologists in a wider range of PRWs as well as in the different subspecialties of ophthalmology, including but not limited to oculoplastics.

Age analysis determined that younger ophthalmologists received higher star ratings and sentiment analysis scores than older ophthalmologists, a finding that was in accordance with those of Smith et al. [11]. This trend does not appear to be unique to the field of ophthalmology, as Tang et al. found similar results among reviews of hand and spine surgeons [14, 15]. However, the geographic location of practice did not yield any statistically significant differences with respect to star ratings or sentiment analysis scores.

Single-word frequency analysis suggested that the highest-rated reviews more often contained words about the ophthalmologist's personality traits such as “friendly,” “caring,” and “kind” than the visit's effectiveness such as “results,” “helpful,” or “efficient,” which was also reflected in the odds ratios of these words as shown in the multivariate logistic regression. Bigram analysis showed similar trends, with “friendly,” “helpful” “kind,” and “caring” being the two most frequent bigrams in positively rated reviews. The presence of “helpful” in the top bigram does, however, indicate the importance of both personality and effectiveness in patient satisfaction. In corroboration, the multivariate analysis underscored that both factors were highly correlated with more positive reviews. Analysis of negative reviews revealed that worst reviews most often involved words that related to timing issues, such as “waiting,” “waited,” and “rushed.” Likewise, Smith et al. noted that ophthalmologists with longer wait times were more likely to receive lower star ratings [11]. Studies that analyzed patient satisfaction through physical surveys such as the Press Ganey survey also found that longer waiting times corresponded to lower satisfaction scores [19, 20]. Bigrams, most often found in the worst reviews, had much lower frequencies than for positive reviews. Still, three of the most frequently used were related to physician availability, namely, “never,” “return;” “refused,” “see;” and “waiting,” “hours.” Lastly, bigram analysis of both the best and worst reviews included references to the staff, underscoring that all these factors that contribute to star ratings were not only the responsibility of the physician but also of every staff member at the practice. Granted, multivariate analysis did not show a statistically significant relationship between “best staff” and positive reviews. However, it is noticeable that “staff” in isolation showed a statistically significant association with positive reviews.

A limitation of PRWs at large is that they inevitably attract the most extreme of opinions. The data suggests that an exceedingly positive or negative experience is more likely to encourage patients to write online reviews than a mediocre experience. For instance, one- and five-star reviews constituted around 96% of the total number of reviews (Table 10). The problem this presents is that it limits the quality of feedback that PRWs can provide ophthalmologists, as it represents a skewed sample of experiences. Consequently, this study advises ophthalmologists to generate their own patient surveys upon completion of the visit. These surveys should be composed of questions that are informed by the single-word, bigram, and multivariate analysis in this study, in order to optimize feedback as patient-centered. By doing so, questions will cater to patients' true concerns and values instead of noninformed surveys that typically cater to the practice's concerns and values. As such, they can offer ophthalmologists a more comprehensive and patient-centric evaluation of their practice than PRWs.

This study itself contains a number of limitations that may warrant follow-up studies. First, it is an observational study that explores only one PRW, Healthgrades, and a broader evaluation of PRWs may yield different and more informed results. It does not include the sentiments of patients who do not have access to the Internet, which may bias against certain groups. Moreover, since the inclusion criteria included ophthalmologists with at least six reviews, it is possible that the ophthalmologists studied were those who either encouraged reviews to their patients or, for positive or negative reasons, attracted a higher number of reviews.

## 5. Conclusion

This study assessed various determinants of ophthalmologist star ratings and their reviews' sentiment analysis scores. These determinants ranged from demographic information such as age, gender, and location to the diction of their patient-written reviews, which revealed the aspects of the ophthalmic care provided that were most or least appreciated by patients. These findings allow us some understanding of patient mindsets when seeking care and thus, should be kept in mind for optimizing practice growth and improving patient care. There was a positive correlation between the VADER score and the Healthgrades star rating, supporting the use of VADER sentiment analysis as a barometer of patient satisfaction. The NLP results of this study suggest that ophthalmologists seeking positive online reviews and presumably increased patient satisfaction should treat patients with an emphasis on being “confident,” “kind,” and “efficient” while also minimizing wait times. This may also be applied to training of staff, scheduling, the creation of practice workflows, and other arenas of practice implementation and governance. For optimization of patient care, ophthalmologists may consider using the single-word, bigram, and multivariate analyzes completed by this study to administer their own patient-surveys. When comparing their reviews and ratings to those of their colleagues, ophthalmologists should note that Healthgrades reviews tend to be biased in favor of younger and male members of the field. However, these findings may also expose selected priorities of these subgroups in patient interactions. Notably, results of the present study's sentiment analysis and word frequency analysis are limited to the Healthgrades database. Future studies may use sentiment analysis to explore multiple PRWs as well as distinguish results between subspecialties of ophthalmology.

## Figures and Tables

**Table 1:** Demographics of ophthalmologists included in analysis.

Demographics	Number
Gender	
Male	626 (55.6%)
Female	499 (44.4%)

Age^*∗*^	
<40	89 (8.9%)
40–49	319 (31.9%)
50–59	310 (31.0%)
>60	282 (28.2%)

Location^*∗*^	
West	136 (12.1%)
Midwest	218 (19.4%)
Southwest	122 (10.9%)
Southeast	431 (38.4%)
Northeast	216 (19.2%)

^*∗*^A number of physicians did not report their age or location and thus were excluded from age or location analyzes.

**Table 2:** Gender analysis based on star rating and review sentiment analysis.

Review type	Male (mean ± SD)	Female (mean ± SD)	*p* values
Star rating (out of 5)	4.61 ± 1.14	4.55 ± 1.22	<0.001
Sentiment analysis (from −1 to 1)	0.645 ± 0.447	0.624 ± 0.465	0.002

**Table 3:** Age analysis based on star rating and review sentiment analysis.

Review type	<40 (mean ± SD)	40–49 (mean ± SD)	50–59 (mean ± SD)	>60 (mean ± SD)	*p* values
Star rating (out of 5)	4.72 ± 0.430	4.51 ± 0.573	4.38 ± 0.709	4.40 ± 0.691	<0.001
Sentiment analysis (from −1 to 1)	0.691 ± 0.150	0.616 ± 0.221	0.586 ± 0.237	0.590 ± 0.230	<0.001

**Table 4:** Tukey HSD of star rating by age groups, FWER = 0.05.

Group 1	Group 2	Mean difference	*p*-adj	Lower	Upper	Reject
40–49	50–59	−0.136	0.063	−0.275	0.0044	False
40–49	<40	0.210	0.051	−0.0005	0.420	False
40–49	>60	−0.114	0.193	−0.257	0.0297	False
50–59	<40	0.345	0.001	0.134	0.556	True
50–59	>60	0.022	0.900	−0.122	0.166	False
<40	>60	−0.323	0.001	−0.537	−0.110	True

**Table 5:** Tukey HSD of sentiment analysis by age groups, FWER = 0.05.

Group 1	Group 2	Mean difference	*p*-adj	Lower	Upper	Reject
40–49	50–59	−0.030	0.441	−0.078	0.018	False
40–49	<40	0.075	0.038	0.003	0.158	True
40–49	>60	−0.026	0.604	−0.075	0.024	False
50–59	<40	0.105	0.001	0.032	0.178	True
50–59	>60	0.004	0.900	−0.046	0.054	False
<40	>60	−0.101	0.002	−0.174	−0.027	True

**Table 6:** Location analysis based on star rating and review sentiment analysis.

Review type	West (mean ± SD)	Midwest (mean ± SD)	Southwest (mean ± SD)	Southeast (mean ± SD)	Northeast (mean ± SD)	*p* values
Star rating (out of 5)	4.48 ± 0.667	4.46 ± 0.694	4.51 ± 0.571	4.50 ± 0.614	4.38 ± 0.692	0.21
Sentiment analysis (from −1 to 1)	0.623 ± 0.230	0.588 ± 0.236	0.634 ± 0.190	0.614 ± 0.220	0.582 ± 0.223	0.12

**Table 7:** Clinically relevant single-word frequency in best and worst reviews.

Best reviews		Worst reviews	
Friendly	1880	Rude	241
Caring	1600	Waiting	237
Kind	1107	Waited	119
Results	1077	Unprofessional	109
Comfortable	727	Rushed	93
Helpful	712	Arrogant	54
Efficient	639	Condescending	49

**Table 8:** Clinically relevant bigram frequency in best and worst reviews.

Best reviews		Worst reviews	
“Friendly,” “helpful”	135	“Never,” “return”	14
“Kind,” “caring”	113	“Refused,” “see”	10
“Friendly,” “efficient”	70	“Staff,” “unprofessional”	9
“Truly,” “cares”	64	“Waiting,” “hours”	9
“Great,” “results”	61	“Billed,” “insurance”	8
“Staff,” “best”	59	“Condescending,” “rude”	8
“Everyone,” “friendly”	51	“Horrible,” “bedside”	7

**Table 9:** Multivariate logistic regression of clinically relevant words ranked by likelihood of inclusion in positive reviews (odds ratio).

Word	5% CI	95% CI	Odds ratio	*p* values
Confident	5.94	23.8	11.9	<0.001
Comfortable	6.61	14.5	9.81	<0.001
Kind	7.08	12.9	9.55	<0.001
Caring	5.57	8.91	7.05	<0.001
Friendly	4.32	6.45	5.28	<0.001
Recommend	3.58	4.42	3.98	<0.001
Efficient	2.72	5.11	3.72	<0.001
Results	2.92	4.52	3.63	<0.001
Clear	2.31	3.71	2.93	<0.001
Warm	1.51	5.09	2.78	<0.001
Staff	2.48	2.99	2.72	<0.001
Listens	1.79	3.96	2.66	<0.001
Best staff	0.272	17.7	2.20	0.461
Wait	0.551	0.675	0.610	<0.001
Return	0.343	0.661	0.476	<0.001
Unprofessional staff	0.0105	3.60	0.194	0.271
Arrogant	0.0328	0.169	0.0746	<0.001
Rude	0.0220	0.0576	0.0356	<0.001
Unprofessional	0.0128	0.0695	0.0298	<0.001

**Table 10:** Spread of star rating scores in all analyzed patient-written reviews.

Star number	Frequency (% of total number)
1	1458 (8.7%)
2	196 (1.2%)
3	140 (0.84%)
4	286 (1.7%)
5	14620 (87.5%)

## Data Availability

All data analyzed in this paper is publicly available through https://www.Healthgrades.com.
